# Beneficial Effects of Omega-3 Polyunsaturated Fatty Acids in Gestational Diabetes: Consequences in Macrosomia and Adulthood Obesity

**DOI:** 10.1155/2015/731434

**Published:** 2015-04-16

**Authors:** Akadiri Yessoufou, Magloire P. Nekoua, Adam Gbankoto, Yohana Mashalla, Kabirou Moutairou

**Affiliations:** ^1^Laboratory of Cell Biology and Physiology, Department of Biochemistry and Cellular Biology, Faculty of Sciences and Techniques (FAST) and Institute of Applied Biomedical Sciences (ISBA), University of Abomey-Calavi, 01 BP 918 Cotonou, Benin; ^2^Department of Animal Physiology, Faculty of Sciences and Techniques (FAST), University of Abomey-Calavi, 01 BP 526 Cotonou, Benin; ^3^School of Medicine, Faculty of Health Sciences, University of Botswana, Private Bag 0022, Gaborone, Botswana

## Abstract

Omega-3 polyunsaturated fatty acids (PUFAs) are increasingly being used to prevent cardiovascular diseases, including diabetes and obesity. In this paper, we report data on the observed effects of omega-3 PUFA on major metabolic disorders and immune system disruption during gestational diabetes and their consequences on macrosomia. While controversies still exist about omega-3 PUFA effects on antioxidant status regarding the level of omega-3 PUFA in diet supplementation, their lipid-lowering effects are unanimously recognized by researchers. Animal studies have shown that omega-3 PUFA contributes to the maintenance of the immune defense system by promoting the differentiation of T helper (Th) cell to a Th2 phenotype in diabetic pregnancy and by shifting the Th1/Th2 ratio from a deleterious proinflammatory Th1 phenotype to a protective anti-inflammatory Th2 phenotype in macrosomia and in adulthood obesity that results from macrosomia at birth. Based on the available evidence, international nutritional and food agencies recommend administration of omega-3 PUFA as triglyceride-lowering agents, for the prevention of cardiovascular disease risk and during human pregnancy and lactation. Furthermore, studies targeting humans are still required to explore application of the fatty acids as supplement in the management of gestational diabetes and inflammatory and immune diseases.

## 1. Introduction

Metabolic disorders as defined by the World Health Organization include disease conditions whose prevalence is reported to be on the increase more so in the developing countries. Based on the results of some epidemiological and clinical investigations in the past few decades, a number of studies have supported the beneficial effects of marine derived omega-3 polyunsaturated fatty acids (PUFAs) in cardiovascular diseases [1–3]. Indeed, low incidence of inflammatory diseases attributed to large consumption of cold water marine fish that contain omega-3 fatty acids has been observed in Greenland Eskimos and Japanese people [4–6]. Since evidence from experimental and clinical studies has proved the beneficial effects of omega-3 fatty acid consumption during diabetes, nutritional strategies have been proposed [7, 8]. Although the mechanism of action of omega-3 fatty acids remains unclear, many reports postulated that the beneficial effects on diabetes and diabetes outcomes may be due to the lipid-lowering action of the fats. However, controversies still exist regarding the beneficial effects of omega-3 PUFA in normal pregnancy or in the treatment and prevention of diabetes during pregnancy and its outcomes on the offspring. The results from most clinical trials performed in type 2 diabetes patients suggest that omega-3 PUFAs have no or marginal effects on metabolic control, while effectively reducing hypertriglyceridemia in these patients [9]. Some authors have recently demonstrated that erythrocyte DHA enrichment with DHA+EPA treatment substantially decreases liver fat percentage in nonalcoholic fatty liver disease patients [10]. Similarly, consumption of lean fish (75–100 g/day) has exhibited beneficial effects by reducing the risk of type 2 diabetes mellitus compared to zero intake in Norwegian women [11]. In contrast, other results have shown that omega-3 PUFA did not provide any benefit on hepatic steatosis and insulin resistance in diabetic patients with nonalcoholic steatohepatitis [12].

Numerous studies have recommended the use of omega-3 PUFA supplementation during human pregnancy and lactation for the prevention of preterm birth, beneficial effects on fetal development, visual and cognitive development, and other functional outcomes of the infants [13–16]. While other authors have found that DHA supplementation (800 mg/day) during the second half of human pregnancy does not reduce the risk of gestational diabetes mellitus or preeclampsia in mothers, others have shown that DHA supplementation can reduce the risk of perinatal death and neonatal convulsions in newborns [17]. Other authors did not find any associations between maternal fatty acid intake or food consumption during human pregnancy and the development of type 1 diabetes in the offspring [18]. Despite these controversial reports on the effects of omega-3 PUFA, guidelines from the Polish Gynecological Association recommended the use of omega-3 PUFA either as supplements or through dietary counseling for women who are planning pregnancy and for patients with normal and/or gestational diabetes and during lactation [19, 20]. Koletzko et al. [21] have published the consensus statement and recommendations of several international research bodies on fatty acids. The adopted conclusions included dietary fat intake in human pregnancy and lactation and recommended that pregnant and lactating women should aim to achieve an average dietary intake of at least 200 mg DHA/day. In addition, since intakes of up to 1 g/day DHA or 2.7 g/day omega-3 long-chain PUFA have been used in randomized clinical trials without significant adverse effects, therefore, women of childbearing age should aim to consume one to two portions of sea fish per week, including oily fish [21]. Moreover, the American Pregnancy Association reports the recommendation of the International Society for the Study of Fatty Acids and Lipids (ISSFAL) that pregnant women should take 300 mg minimum to support themselves and the fetus for DHA requirements on a daily basis [22, 23]. The Institute of Medicine Food and Nutritional Board has developed what is considered as the recommended minimum adequate intake levels for the omega-3 PUFA group. The recommended adequate intakes for omega-3 PUFA are 1.3 g/day for nursing women, 1.1 g/day for adult women, 1.4 g/day for pregnant women, 1.3 g/day for girls ages 14 and above, 1.6 g/day for boys ages 14 and above and adult men, 0.5 g/day for infants, 0.7 g/day for children (1 to 3 years old), and a dosage of 0.9 g/day for children (4 to 8 years old) [22, 23].

The scientific evidence for cardioprotective effects of food sources of omega-3 PUFA, eicosapentaenoic acid (EPA), and docosahexaenoic acid (DHA), beyond the effect of changes in serum lipid profiles, has been recognized by the American Heart Association (AHA) Dietary Guidelines. The AHA recommended consumption of at least two servings of fish per week to confer cardioprotective effects [24]. In the same line the US Food and Drug Administration has approved administration of omega-3 fatty acids only as triglyceride-lowering agents in patients with hypertriglyceridemia [24]; and some European regulatory agencies have approved the use of omega-3 for the treatment of cardiovascular risk [9]. The aim of the present paper is to review data on the beneficial effects of omega-3 PUFAs on major metabolic disorders and immune system disruption observed during gestational diabetes and macrosomia. Details of outcomes of maternal diabetes in pregnancy on offspring have been reviewed elsewhere [25] and, therefore, will only be briefly discussed before focusing on beneficial effects of PUFA during gestational diabetes and evaluating the consequences on the macrosomia in newborns that become obese in adulthood.

## 2. Major Metabolic Complications during Gestational Diabetes and Macrosomia

Gestational diabetes mellitus (GDM, which refers to diabetes only during pregnancy) and obesity during pregnancy are both complications which significantly influence the development of offspring during fetal life and postnatal. Indeed, animal and human studies indicated that fetuses from mothers with gestational diabetes are at high risk of developing fetal macrosomia [26, 27], and they are prone to adverse side effects strongly associated with prematurity, birth trauma, respiratory distress syndrome, and fetal death [28]. Effectively, our observations are in agreement with previous epidemiological and clinical trials that have shown that either preexisting maternal diabetes (type 1 and type 2) or GDM appears to be important risk factor for fetal overnutrition and macrosomia [29–32].

Several modes exist for inducing experimental maternal diabetes with streptozotocin in animal models and the consequences on fetus and adult progeny are variable with each model [33, 34]. The streptozotocin, when administered at a high single dose, induces diabetes by the direct toxic effects on pancreatic *β*-islet cells [33]. The fetus is confronted with severe intrauterine hyperglycemia which induces fetal islet hypertrophy and *β*-cell hyperactivity and may result in early hyperinsulinemia [34]. The increased insulin secretion dramatically and rapidly decreases due to the overstimulation of fetal *β* cells which are depleted of insulin granules, resulting in fetal hypoinsulinemia [33, 34]. The growth of fetal protein mass is then suppressed, leading to fetal microsomia (small birth weight) [33]. Postnatal development is affected and retarded, and the offspring remain small at adulthood but develop insulin resistance [33, 35].

The animal model reported in this review concerns mild streptozotocin-induced type 1 diabetic pregnancy which also leads to macrosomia in newborns [36, 37]. Streptozotocin, administered at low doses during 5 consecutive days, induces mild type 1 diabetes, following a T-lymphocyte-dependent process, an autoimmune destruction of pancreatic *β* cells, mediated by both CD4^+^ and CD8^+^ T cells [38, 39] and this represents a good model of diabetes development for several reasons [38, 40, 41]. When the streptozotocin is administrated at five low doses, starting on day 5 of gestation to preserve gestation in pregnant rats, [36] the infiltration of pancreatic islet *β*-cells by autoreactive T lymphocytes is observed two days after the last injection [38]; the hyperglycemia occurs one week (7 days) after the last injection. Diabetes (hyperglycemia) becomes maximal around 10-11 days after the last STZ injection (i.e., second trimester of gestation) [38–41] and it persists after delivery [25]. We have previously shown that the progenies of pregnant diabetic rats are prone to develop macrosomia at birth, obesity, type 2 diabetes, and impaired glucose tolerance in adulthood [27, 42].

Studies in humans with GDM revealed that diabetes determined by oral glucose tolerance test according to the criteria of the World Health Organization, as reviewed by the International Association of Diabetes and Pregnancy Study Groups (IADPSG) based on the Hyperglycemia and Adverse Pregnancy Outcomes (HAPO) Study [43], appeared at second or third trimester of pregnancy [28, 29], as we described elsewhere [25]. GDM patients are hyperglycemic and hyperinsulinemic at the diagnosis of the disease [28, 29], reflecting a decrease in insulin sensitivity in diabetic pregnant women [44]. Maternal diabetes is characterized by an increased placental transport of glucose and other nutrients from the mother to the fetus, resulting in macrosomia [33]. Convincing evidence from our studies and others has shown that either preexisting diabetes (type 1 and type 2 diabetes) or GDM (diabetes only during pregnancy) appears to be important risk factor for fetal overnutrition and macrosomia in newborns and for the development of diabetes and adulthood obesity that results from macrosomia [26, 29–32, 44–46]. Macrosomia, the most commonly reported effect of maternal diabetes in newborns [45], is usually defined in humans as birth weight above either 4 kg or birth weight above the 95th percentile of the gestational age. Babies from GDM patients whose birth weight was 2.0 SD greater than the mean birth weight of control infants were considered as macrosomic babies [26, 46, 47]. The risk of diabetes in the offspring of type 2 diabetes genitors is significantly higher when the mother rather than the father is diabetic [31]. Moreover, the risk of insulin resistance is higher in children of mothers with GDM (diabetes only during pregnancy) than in children from mothers developing diabetes after pregnancy [48]. Therefore, diabetic pregnancy appears to induce macrosomia that results in obesity in adulthood and these pathologies are associated with several metabolic disorders, implicating lipid metabolism, altered antioxidant status, and disrupted immune defense system.

### 2.1. Effects of Maternal Diabetes on the Lipid Metabolism: Implication in Macrosomia

Regarding metabolic processes, maternal diabetes induces alterations in the lipid metabolism which contribute to macrosomia in newborns. Indeed, we have previously shown in animal and human studies that diabetic pregnancy induces maternal hyperlipidemia which predisposed the fetus to macrosomia [26, 27, 42]. In fact, high levels of triglyceride in the maternal circulation of diabetic rats tend to create a steep concentration gradient across the placenta which accelerates the transport and deposition of the lipids in fetal tissues [49]. In addition, maternal hyperglycemia also leads to fetal hyperglycemia, which stimulates pancreatic islet cells and induces fetal hyperinsulinemia in animals and humans [26, 33–35, 37, 42, 46, 47]. Animal studies also showed that in macrosomic newborns hypertriglyceridemia exists and persists with age and is linked to the development of insulin resistance and hyperlipogenesis at adulthood [37]. Our observations are confirmed by several recent studies which have shown that maternal diabetes in human and rat is associated with increased risk of hyperlipidaemia [50–54] and metabolic syndrome and type 2 diabetes in the offspring [53, 54].

### 2.2. Effects of Maternal Diabetes on the Antioxidant Status: Implication in Macrosomia

In human studies as well as in experimental animal models, we have observed that maternal diabetes significantly alters the total antioxidant status as demonstrated by decreased antioxidant molecules (vitamins A and E), enzyme activities (superoxide dismutase (SOD), glutathione peroxidase (GSH-Px), and glutathione reductase (GSSG-Red)), and increased serum thiobarbituric acid-reactive substances (TBARS) [27, 46]. The altered antioxidant system is also observed and persists with age in the macrosomic rat and human newborns that became obese adults [27, 46]. These observations are recently supported by several investigators who have observed increased oxidative stress in gestational diabetic women and animals [55–58] and their infants [59] and rat adult offspring [60]. In fact, our findings suggest that there is an increased oxidative stress in diabetic pregnant women and rats and their adult obese offspring that were macrosomic as newborns [27, 46], in agreement with the results of previous studies [61–64].

### 2.3. Effects of Maternal Diabetes on the Immune System: Implication in Macrosomia

**Figure 1:**
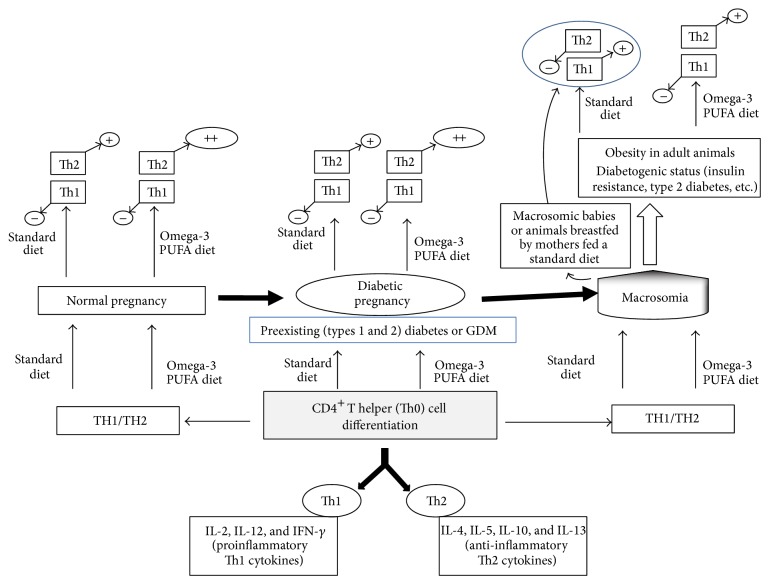
Effects of omega-3 PUFA diet on Th1 and Th2 dichotomy in animal diabetic pregnancy: implication in macrosomia. Naïve CD4^+^ T helper (Th0) cells can be differentiated into either Th1 cells, producing proinflammatory cytokines (IL-2, IL-12, and IFN-*γ*), or Th2 cells, secreting anti-inflammatory cytokines (IL-4, IL-10, IL-5, and IL-13). Human and animal studies show that, in normal pregnancy as well as in diabetic pregnancy, the Th1/Th2 balance is shifted towards a protective Th2 phenotype. Feeding omega-3 PUFA diet to rats, in normal pregnancy as well as in diabetic pregnancy, enhances the increase of Th2 cytokines. In contrast, the Th1/Th2 balance is shifted towards a proinflammatory Th1 phenotype in macrosomic newborns as well as in adult obese animals that were macrosomic as newborns and the omega-3 PUFA diet shifts the ratio to an anti-inflammatory Th2 phenotype in adult obese animals. Th: T helper cells; GDM: gestational diabetes mellitus; PUFA: polyunsaturated fatty acid; (+): upregulation; (−): downregulation. Data are from the studies carried out by Khan et al., J Autoimmun, 2006 [65].

In animal as well as in human studies, the immune system is also shown to be modulated during maternal diabetes which induces macrosomia in newborns. Several studies have implicated a pathological role of the immune system and inflammation in type 1 diabetes, type 2 diabetes, and GDM. Indeed, T cell-derived cytokines are involved in the autoimmune destruction of pancreatic islet cells leading to type 1 diabetes [38] while type 2 diabetes is associated with a generalized activation of the innate immune system, in which there is a chronic, cytokine-mediated state of low-grade inflammation [66–68]. Normal pregnancy or pregnancy complicated with diabetes is known to influence T helper cell differentiation. Evidence from our studies revealed that in normal pregnancy Th1 cytokines are downregulated whereas Th2 cytokines are upregulated in animals as well as in humans [65, 69] (Figure 1). Our observations were in agreement with the results of previous studies [70, 71]. Interestingly, we have observed that in diabetic pregnancy Th1 cytokines decrease and IL-10, a Th2 cytokine, increases [26, 65, 69] as presented in Figure 1. Therefore, evidence has shown that Th2 cytokines may be beneficial for successful pregnancy in diabetic animals and GDM patients. Indeed, the shift of Th1/Th2 ratio to a protective Th2 phenotype during pregnancy has been shown to promote humoral immunity with high production of antibodies which contribute to the fight against infections during pregnancy and offer passive immunity to fetus [72]. However, animal and human studies have shown that, in macrosomic newborns and obese adult animals that were macrosomic as newborns, the Th1/Th2 balance is shifted to a proinflammatory Th1 phenotype [26, 65] (Figure 1). This upregulated-Th1 profile in obese adult animals that were macrosomic as newborns may confer to these animals a potential “diabetogenic status,” as revealed by the hyperglycemia and hyperinsulinemia observed in these animals in the adulthood [42, 65].

## 3. Effects of Omega-3 PUFA in Gestational Diabetes: Incidence on Macrosomia and Lipid Metabolism

We have previously examined in animal model the effects of omega-3 PUFA on the incidence of macrosomia in diabetic pregnancy in rats [27, 42, 65]. The model of diabetic pregnancy was established through administration of five low doses of streptozotocin to pregnant Wistar rats starting on day 5 of gestation as described above [27, 37, 38, 42, 65]. Pups from diabetic pregnant rats whose birth weights were 1.7 SD greater than the mean birth weight of the control pups were considered as macrosomic newborns [27, 37, 38, 42, 65]. We observed that 62% to 75% of pups of diabetic pregnant rats were macrosomic at birth [27, 42, 65]. These macrosomic newborns were hyperglycemic at birth and, when compared to offspring of control rats, they maintained an accelerated weight gain and become obese at adulthood (3 months of age) [27, 42, 65]. Interestingly, we observed that omega-3 PUFA diet consumption significantly reduced the incidence of gestational diabetes on macrosomia by decreasing the rate of macrosomic newborns by 16–25% [27, 65]. However, omega-3 PUFA diet did not show any effect on the hyperglycemia of macrosomic newborns that become obese at adulthood [27].

In the animal model, while diabetic pregnancy associated with hyperlipidemia [27, 49, 73] has been reported to induce hypercholesterolemia and hypertriglyceridemia in adult obese offspring from macrosomic newborns born to diabetic animals [27, 37, 74, 75], our studies have demonstrated that omega-3 PUFA diet significantly reduced the levels of cholesterol and triglyceride in diabetic pregnant animals and attenuated hyperlipidemia in their adult obese offspring from macrosomic newborns [27, 42, 65]. The hypolipidemic effects of omega-3 PUFA diet have also been demonstrated in animals [27, 42, 65] as the same findings have previously been reported in human studies by other researchers [4, 7, 8]. The hypocholesterolemic effects of omega-3 PUFA diet have been observed not only in the serum but also in the liver of diabetic pregnant animals and their adult obese offspring from macrosomic newborns. The findings suggest a decrease in cholesterol synthesis or increased cholesterol excretion into bile. It has been reported that fish oil induces changes in cholesterol metabolism in rat liver leading to an increase in the biliary excretion of cholesterol [76]. In our study, it also has been established in rat dams and their breastfed macrosomic pups that become obese in the adulthood that dietary intake of omega-3 PUFA induces a large increase in plasma omega-3 PUFA levels followed by a large decrease in omega-6 PUFA (LA and AA in particular) [77], in agreement with previous results [78]. Thus, we concluded that omega-3 PUFA exerts its beneficial effects on lipid metabolism observed in diabetic pregnant animals and their macrosomic newborns that become obese adults by attenuating the hyperlipidemia associated with these pathologies.

## 4. Antioxidant Effects of Omega-3 PUFAs during Gestational Diabetes and Macrosomia

In human studies as well as in animal models, we and several authors have previously reported that diabetes, diabetic pregnancy, macrosomia, and adulthood obesity that results from macrosomia are associated with increased oxidative stress related to decreased antioxidant molecules (vitamins A and E), decreased antioxidant enzyme activities (superoxide dismutase (SOD), glutathione peroxidase (GSH-Px), and glutathione reductase (GSSG-Red)), and increased serum thiobarbituric acid-reactive substances (TBARS) [25, 25, 27, 61–64, 79, 80]. High blood glucose has been shown to induce an oxidative stress which in turn induces the production of highly reactive oxygen species toxic to cells particularly the plasma membranes where the radicals interact with the lipid bilayer [61]. Under normal conditions, endogenous antioxidant enzymes and vitamins are responsible for detoxification of the deleterious oxygen radicals. Thus, treatment with antioxidants may prevent or reverse the abnormalities associated with diabetes and its complications. Some studies have reported that dietary supplements with vitamins and minerals prevent or at least attenuate the organic deterioration caused by an excessive oxidative stress associated with diabetes in humans and animals [81, 82]. There is a general notion that omega-3 PUFA might deteriorate antioxidant capacity. Nonetheless, no consensus has been reached on this subject as shown in Table 1.

It has been argued that excessive intake of omega-3 PUFA may affect antioxidant status [8, 83–86] and enhance the susceptibility to oxidative damage. While some investigators [87–89] could not find any changes in the antioxidant status in humans and rats treated with omega-3 fatty acid-rich diet, we and other researchers have demonstrated that treating diabetic patients [90] or gestational diabetic rats and their adult obese offspring that were macrosomic as newborns [27] with omega-3 fatty acids significantly improves their antioxidant status. The details of fatty acid compositions of control and omega-3 PUFA diets used in our previous studies [27, 65, 91] are presented in Table 2. From the results presented in Table 1, we concluded that a moderate level of omega-3 PUFA dietary intake could be beneficial for improving the antioxidant status. This argument is supported by the findings that dietary fish oil modulates the composition of plasma membrane phospholipids by increasing omega-3 PUFA contents (EPA and DHA in particular) at the expense of arachidonic acid (AA, an omega-6 PUFA) levels [88]. Similarly, we have previously reported that feeding an omega-3-enriched diet to animals leads to an increased incorporation of EPA and DHA into the plasma membrane phospholipids of T lymphocytes and a decrease in arachidonic acid level (Table 3) [77, 91]. Our findings have been supported by other researchers [92–94]. Hence, we have concluded that omega-3 fatty acids influence T cell activity by being incorporated into their plasma membranes (see Table 3). The incorporation of omega-3 PUFA into the cell plasma membranes may diminish or counterbalance the negative effects of AA (n-6 PUFA) on antioxidant status and consequently modulate cell activation [92].

The balance between omega-3 and omega-6 fatty acids may also markedly affect cell metabolism. Evidence in the literature shows that essential fatty acids influence the physical properties of cell membranes in terms of fluidity and permeability, activity of membrane receptors, enzymes and ion channels, and cell response to various stimuli through the production of secondary messengers [92]. Therefore, the beneficial effect of omega-3-diet on antioxidant status likely involves DHA and EPA because EPA is known to give rise to eicosanoids of omega-3 fatty acid series which exert opposite effects to those of omega-6 series derived from linoleic acid (LA) and arachidonic acid (AA). In addition, EPA may also be converted into DHA which, along with dietary DHA, may further contribute to the beneficial effects. It has also been shown that DHA may give rise to some discovered derivatives like docosatrienes or resolvins which exert beneficial effects on the antioxidant status [95]. Similarly, the fatty acids have been shown to modulate cell signaling mechanisms via their incorporation in the plasma membrane phospholipids [96]. Although the exact mechanism by which EPA/DHA exert antioxidant action is not well understood, Das et al. [97] have suggested that EPA/DHA supplementation inhibits free radical generation and suppresses lipid peroxidation and NO synthesis in patients with nephritic syndrome. This finding suggests that EPA or DHA may be involved in scavenging of free radicals and NO. In support of the finding, Yazu et al. [93] reported that in aqueous micellar dispersions composed of methyl esters of EPA or linoleate (LA), the oxidizability of the methyl ester of EPA (omega-3) was lower than that of methyl linoleate (omega-6). The EPA micelle had ≥2 molecules of oxygen in the peroxyl radical while the linoleate micelle had only one molecule suggesting that EPA is more polar than linoleate and the oxygen species polar radicals may migrate from the lipophilic core of the micelle to the polar surface. Due to this migration, an environment was created that favoured the termination and reduced the propagation of oxidation reactions [84]. This ability of EPA to behave as peroxyl and free radical scavenger is one of the mechanisms which may be used to explain the antioxidant properties of the fatty acid. With regard to the antioxidant properties of EPA/DHA, it has recently been proposed that DHA inhibited more efficiently than EPA the protein degradation by regulating NF*κ*B (Nuclear Factor kappa B) signaling pathway in mouse C2C12 myotubes through activating PPAR*γ* gene expression [98]. In addition, other authors have found that DHA and genistein exert complementary actions whilst genistein is antagonistic to arachidonic acid (an omega-6 PUFA) for controlling prostaglandin E_2_ production as well as invasiveness of MDA-MB-231 human breast cancer cells in culture by modulating the level of NF*κ*B expression [99].

## 5. Effects of Omega-3 PUFA on the Immune System in Gestational Diabetes: Implication in Macrosomia

As stated above, the immune system plays a preponderant role in the pathogenesis of maternal diabetes in pregnancy and macrosomia and both pathologies involve T cell activation. Many investigators have shown interest in the effects of omega-3 fatty acids on several diseases including diabetic pregnancy and obesity. It has been established that omega-3 PUFAs exert immunosuppressive effects [92, 94]. Being immune modulator agents, omega-3 PUFAs are thought to play an important role in the modulation of immune cell activation by exerting action through Th1/Th2 dichotomy in diabetic pregnancy and macrosomia. Consequently, the fatty acids are being used in the management of diabetes mellitus in human beings [100] and experimental models [94] and also in several inflammatory and autoimmune diseases including rheumatoid arthritis and multiple sclerosis [101]. Physiologically, n-3 PUFA suppresses mitogen-stimulated proliferation of lymphocytes isolated from lymph nodes [94]. It has also been shown that dietary EPA and DHA are equipotent in inhibiting IL-2 production in rodents [102, 103]. The production of IFN-*γ* is also decreased by the fatty acids [104]. Hence, it can be argued that the exhibited potential action of the fatty acids on cytokine secretion [102] is attributed to eicosapentaenoic acid (EPA) and docosahexaenoic acid (DHA) which appear as the most potent immunomodulators of omega-3 PUFA family.

With regard to the effects of omega-3 PUFA on diabetic pregnancy, previous studies have established that feeding omega-3-enriched diet to pregnant healthy animals potentiates the increase of the Th2 phenotype in the lymphoid organ (spleen) and peripheral blood of the animals on standard diet [65, 105]. In diabetic pregnancy however, omega-3-enriched diet increases Th2 cytokines and decreases Th1 cytokines at both expressing and circulating levels [65].

In animal and human studies, a shift of Th1/Th2 balance to a proinflammatory Th1 phenotype has been observed in macrosomic newborns and adult obese animals that were macrosomic as newborns of diabetic dams [26, 65]. As compared to animals fed on a standard diet, feeding the omega-3 PUFA enriched diet to adult obese rats from macrosomic newborns significantly diminishes the mRNA expression of Th1 cytokines and increases that of IL-4 but not that of IL-10 [65] (Figure 1). While the circulating high concentrations of IFN-*γ* observed in adult obese rats from macrosomic newborns are lowered by omega-3 PUFA diet, the IL-4 level observed in the animals is increased by the omega-3 PUFA diet (Figure 1). These findings suggest that omega-3 PUFA diet exerts beneficial effects in obese rats from macrosomic newborns by significantly shifting the Th1/Th2 (IFN-*γ*/IL-4) ratio to a Th2 phenotype [65] (Figure 1). However, the omega-3 PUFA diet could not significantly influence glycaemia in macrosomic newborns and obese rats, suggesting that macrosomia may be a multifactorial pathology.

## 6. Conclusion

Gestational diabetes mellitus and macrosomia that results in adulthood obesity are pathologies associated with several metabolic disorders, implicating lipid metabolism, altered antioxidant status, and disrupted immune defense system. Based on the evidence available in animal studies, feeding omega-3 PUFA diet not only decreases the high rate of macrosomia induced by diabetic pregnancy but also exerts a lipid-lowering action in both pathologies. Omega-3 PUFA also contributes to the protection against oxidative stress during gestational diabetes and adulthood obesity that results from macrosomia and restores the immune defense system disrupted by diabetes. The protection is by enhancing Th2 phenotype in diabetic pregnancy and therefore shifting the Th1/Th2 phenotype from a deleterious proinflammatory Th1 phenotype to a protective anti-inflammatory Th2 phenotype in offspring that were macrosomic at birth and became obese in the adulthood. Further studies targeting humans are recommended to further explore application of the fatty acids as supplements in the management of gestational diabetes and inflammatory and immune disease conditions.

## Figures and Tables

**Table 1:** Effects of omega-3 fatty acids on antioxidant status as reported by various investigators. This table is adapted from our previous study, Yessoufou et al., Int. J Obesity, 2006 [27].

Antioxidant status	Species	Omega-3 PUFA level in the diet	References
Decreased	Diabetic rats	10% of diet (considered as excessive)	Cho and Coi, 1994 [83]
Decreased	Healthy humans	EPA: 2.5 g/day; DHA: 1.8 g/day	Wander and Du, 2000 [84]
Decreased	Healthy humans	6.26 g/day for 6 weeks	Allard et al., 1997 [85]
Decreased	Patients with myocardial infarction	850–882 mg/day (EPA + DHA) for 1 year	Grundt et al., 2003 [86]
Decreased	Diabetic rats	Fish oil	Yilmaz et al., 2002 [8]
Unchanged	Healthy humans	4 g/day (n-3) PUFA for 5 weeks	Hansen et al., 1998 [87]
Unchanged	Rats	n-3 fatty acid-rich diet (fish oil)	Ando et al., 1998 [88]
Unchanged	Hyperlipidemic patients	4 g/day (DHA or EPA)	Nordøy et al., 1998 [89]
Improvement	Diabetic humans	EPA: 1.08 g/day; DHA: 0.72 g/day	Kesavulu et al., 2002 [90]
Improvement	Diabetic rats	2.1% of diet	Yessoufou et al., 2006 [27]

**Table 2:** Fatty acid composition of control and omega-3 PUFA diets.

Fatty acids	Control diet (mg/g)	EPAX diet (mg/g)
C14:0	0.4	0.4
C16:0	5.1	2.1
C18:0	3.9	1.7
C18:1	18.5	9.1
C18:2n-6	21.3	11.2
C18:3n-3	0.83	0.5
C20:4n-6 (AA)	ND	0.9
C20:5n-3 (EPA)	ND	22.2
C22:6n-3 (DHA)	ND	2.0
**Total fatty acids**	**50.0**	**50.0**
∑n-6 PUFA	21.30	12.06
∑n-3 PUFA	0.83	24.59
(n-6)/(n-3)	25.80	0.49
(n-3)/(n-6)	0.04	2.04
∑SFA	9.40	4.26
∑PUFA	22.13	36.65
∑MUFA	18.50	9.07
PUFA/SFA	2.35	8.60

ND = not detectable. This table is adapted from our previous studies [27, 65, 91].

The chemical composition of control diet was as follows (g/kg dry diet): starch, 587; casein, 200; cellulose, 50; sucrose, 50; mineral mix, 40; vitamin mix, 20; DL-methionine, 3; vegetable oil-Isio-4 (Lesieur, Neuilly-sur-Seine, France), 50. Total oil represented 5% of the diet. In the omega-3 PUFA diet, half of the vegetable oil-Isio-4 was replaced by EPAX-7010 (the omega-3 PUFA oil). The vegetable Isio-4 oil contained the following: 47.2 mg/g 18:2 (n-6); 1.7 mg/g total (n-3); and 40.2 mg/g monounsaturated fatty acids (largely 18:1). EPAX-7010 oil, in the form of ethyl ester, contained approximately 85% (n-3) PUFA, that is, EPA, 70%, DHA, 12%, and *α*-tocopherol, 2.1 to 3.2%. It means that EPAX oil represented 2.5% of the diet. Since the omega-3 PUFA consisted of 85% of the 2.5% EPAX oil, the total n-3 PUFA represented only 2.1% of the total diet. After diets' preparation, the lipids from diets were extracted according to the method described in Yessoufou et al., 2006 [27], and then transmethylated by BF3/methanol after saponification, and fatty acids were analysed by gas liquid chromatography.

**Table 3:** Fatty acid composition of plasma membrane phospholipids of T lymphocytes purified from the spleen of mice fed on standard diet or omega-3-enriched diet, adapted from our previous study, Yessoufou et al., J Lipid Res., 2009 [91].

Fatty acids (% of total)	Cells from mice fed standard diet	Cells from mice fed omega-3 PUFA diet
C16:0	1.99 ± 0.31	2.16 ± 0.39
C16:1	27.01 ± 0.37	27.37 ± 0.20
C18:0	18.79 ± 0.25	18.31 ± 0.67
C18:1	13.15 ± 0.56	12.78 ± 0.75
C18:2n-6	11.12 ± 0.75	10.19 ± 0.11
C20:4n-6 (AA)	25.67 ± 0.98	13.12 ± 0.28^*^
C20:5n-3 (EPA)	0.41 ± 0.05	6.09 ± 0.15^*^
C22:6n-3 (DHA)	1.77 ± 0.06	9.99 ± 0.41^*^

Cells were purified from the spleen of mice fed the standard diet or omega-3-diet for 6 weeks. Values are mean ± SEM, *n* = 10 mice per group of diet. ^*^ *P* < 0.01: significant differences between omega-3-diet group and standard diet group. The lipids from cells were extracted according to the method described in the following reference: Yessoufou et al., J Lipid Res., 2009 [91]. Phospholipids were separated from silica gel by thin layer chromatography, using the following solvent: chloroform/methanol/acetic acid at 35 : 14 : 2.7 (v/v/v). After scraping off, the phospholipid fractions were transmethylated by BF3/methanol after saponification, and fatty acids were extracted and further analyzed by gas liquid chromatography. Analysis of fatty acid peaks was achieved with reference to the internal standard by using DELSI ENICA 21 integrator (Delsi Nermag, Rungis, France).

## Abbreviations

GDM: Gestational diabetes mellitus
PUFA: Omega-3 polyunsaturated fatty acids
CD: Cluster of differentiation
IL: Interleukin
IFN: Interferon
AA: Arachidonic acid
LA: Linoleic acid; eicosapentaenoic acid (EPA); and docosahexaenoic acid (DHA)
Th cells: T helper cells
TBARS: Thiobarbituric acid-reactive substances
TG: Triglyceride
TC: Total cholesterol
SOD: Superoxide dismutase
GSH-Px: Glutathione peroxidase
GSSG-Red: Glutathione reductase.
